# COVID-19 Infection Increases Mortality and Complications in Patients With Neck of Femur Fracture

**DOI:** 10.7759/cureus.22264

**Published:** 2022-02-15

**Authors:** Nuthan Jagadeesh, Sachindra Kapadi, Venkatesh Deva, Deepak Channabasappa, Debbie Shaw

**Affiliations:** 1 Trauma and Orthopaedics, Wrightington, Wigan and Leigh NHS Foundation Trust, Wigan, GBR

**Keywords:** 30-day mortality, 90-day mortality, impact of covid-19, fracture neck of femur, covid 19

## Abstract

Hip fractures commonly occur in elderly patients with multiple comorbidities. Contracting coronavirus disease 2019 (COVID-19) when healing from hip fractures places the patients at a higher risk of respiratory compromise and death. This study aimed to compare the 30- and 90-day mortality rates of patients with hip fracture with and without COVID-19. The secondary aim was to determine the impact of COVID-19 on the parameters of morbidity such as health complications and length of hospital stay. All patients with hip fractures who presented to our hospital between March and December 2020 were classified into one of two subgroups: those with a clinical and/or laboratory diagnosis of COVID-19 and those without. Patient demographics, American Society of Anesthesiologists score, Nottingham Hip Fracture Score, Charlson Comorbidity Index, complications, length of stay, and 30- and 90-day mortality rates were measured in patients with hip fractures with and without a clinical diagnosis of COVID-19. We found that COVID-19 infection independently increased the 30- and 90-day mortality rates, respiratory complications, and length of hospital stay in patients with hip fractures. This is the first study to report the 90-day mortality of COVID-19 infection in such patients.

## Introduction

The World Health Organization (WHO) declared the outbreak of coronavirus disease 2019 (COVID-19) as a pandemic on March 11, 2020. This global catastrophe has transformed orthopedic practice. Elective orthopedic procedures were deprioritized as governments worldwide devoted time and resources toward the treatment of patients with COVID-19. Moreover, bans placed on non-essential travel and encouragements to stay at home reduced the number of trauma admissions to hospitals in the United Kingdom (UK) [1]. Ogliari et al. indicated decreased non-hip fragility fractures, such as distal radius, proximal humerus, and ankle fractures, due to fewer outdoor falls [2]. However, UK hospitals identified that changes in social behavior and mobility during the pandemic did not affect the number of hip fragility fractures [3,4].

UK's National Hip Fracture Database reported 67,302 hip fractures in 2019 [5,6]. The treatment of hip fractures constitutes a majority of the National Health Service budget, with an increase in hospital costs the first year following a hip fracture [7]. Fragility hip fractures typically occur in elderly individuals aged >60 years, up to 60% of whom have at least one comorbidity [8]. The mortality rates for these fractures at 30 days and one year are 6.1% and 33%, respectively [5,6]. However, elderly individuals infected by COVID-19 have a 15.4-fold increased risk of mortality than younger individuals [8]. Additional comorbidities, including diabetes mellitus and respiratory conditions, further increase the risk of death [9]. The mechanism by which COVID-19 affects the mortality of hip fractures is poorly understood. The 30-day mortality in such cases is 30%-35% [10-12]. However, these studies were performed over one to two months, whereas the COVID-19 pandemic had disrupted services throughout 2020. Short-term studies do not truly indicate actual mortality as many patients die after 30 days. Therefore, 90-day mortality may be a better metric for such patients.

The primary aim of our study was to evaluate the independent impact of COVID-19 infection on the 30- and 90-day mortality rates of patients with hip fractures and compare them with those of uninfected patients. This work also explored how COVID-19 affects morbidity parameters, including complications and length of hospital stay, in these patients with fragility hip fractures.

## Materials and methods

This retrospective study was conducted at the Royal Albert Edward Infirmary, Wigan, UK, from March 1 to December 31, 2020. All patients who were admitted to our hospital with hip fractures during the study period were included. Diagnostic criteria for COVID-19 included a positive result of quantitative reverse transcription-polymerase chain reaction (RT-PCR) testing of respiratory tract samples, clinical symptoms suggestive of COVID-19, and or chest abnormalities on computed tomography (CT) indicative of COVID-19. The types of fractures included in this study were those classified as hip fractures by the National Hip Fracture Database, which included displaced and undisplaced intracapsular, intertrochanteric, and subtrochanteric fractures [5,6]. Patients with polytrauma and femoral shaft and distal femur, open, isolated greater trochanter, periprosthetic, or pelvic fractures were excluded.

Demographic variables like age, sex, ethnicity, and type of residence (care home or own home) were collected. Data on the type of fracture, intervention, the timing of surgery, and length of hospital stay were also recorded. Nottingham Hip Fracture Score (NHFS), American Society of Anesthesiologists (ASA) score, and Charlson Comorbidity Index (CCI) were calculated for each patient. The primary outcome parameters were 30- and 90-day mortality rates and the secondary outcome parameters were complications and the length of hospital stay. Surgery was considered as delayed if performed >36 hours after admission. COVID-19 tests were administered during admission and at regular intervals during the hospital stay. Moreover, the time when a patient acquired COVID-19, before admission or during the hospital stay, was noted.

All patient characteristics were summarized descriptively. Mean ± standard deviations were reported for continuous variables. Categorical data were summarized and presented via diagrams. Chi-square (χ2) test was used to analyze the association between two categorical variables. An unpaired t-test was used to determine the difference of the means of analysis variables between two independent groups. Cox regression and Kaplan-Meier analyses were used to compare the mortality rates between groups. A p-value of <0.05 indicated statistical significance. Data were analyzed using SPSS software v.23 (IBM Corp., Armonk, NY) and Microsoft Office 2007 (Microsoft Corporation, Redmond, WA).

## Results

The study period was from March to December 2020 as the first case of COVID-19 in the UK was detected in the month of March. Overall, 278 patients with neck of femur fractures were admitted to our hospital during the study period, and 51 of these were diagnosed with COVID-19 via laboratory testing or clinical/radiological symptoms suggestive of COVID-19. There was no difference in the mean ages of the COVID-19-positive (80.7 ± 10.9 years) and the COVID-19-negative (78.8 ± 11.4 years) groups (p = 0.277). Approximately 20.9% of patients in the “positive group” and 23.8% of those in the “negative group” were care home residents (p > 0.551). There were no gender-based differences between the two groups (p = 0.389). The majority of patients were females, accounting for 64.7% and 70.8% of the COVID-19-positive and COVID-19-negative groups, respectively (Table 1).

**Table 1:** Comparison of various parameters between COVID-19-positive group with COVID-19-negative group. Note: * p-value significant at 5% level of significance (p < 0.05). ASA: American Society of Anesthesiologists score; AMTS: Abbreviated Mental Test Score.

Parameters	Number of cases	COVID-19-positive group (n = 51)		COVID-19-negative group (n = 223)		P-value
		N	%	N	%	
Age						
Mean	274	80.7 ± 10.9		78.8 ± 11.4		0.277
Sex						
Male	83	18	35.3	65	29.1	0.389
Female	191	33	65.7	158	70.9	
Type of fracture						
Intertrochanteric fracture	79	15	29.4	64	28.7	0.096
Neck of femur	192	34	66.7	158	70.9	
Subtrochanteric	3	2	3.9	1	0.45	
Type of operation						
Screw	5	1	1.96	4	1.79	0.229
Dynamic hip screw	92	13	25.49	79	35.42	
Hemiarthroplasty	136	27	52.94	109	48.87	
Intramedullary nail	16	6	11.76	10	4.48	
Not operated	11	3	21.56	8	3.58	
Total hip replacement	14	1	1.96	13	5.82	
Nottingham hip fracture score						
Mean	274	4.9 ± 1.7		4.7 ± 1.7		0.526
ASA						
Mean	274	3 ± 0.6		3 ± 0.7		0.786
1	8	2	3.92	6	2.69	0.350
2	36	3	5.88	33	14.79	
3	178	38	74.5	140	62.78	
4	52	8	15.68	44	19.73	
Charlson Comorbidity Index (mean)	274	5.2 ± 2.1		4.7 ± 2.2		0.138
Myocardial infarction	37	7	13.72	30	13.45	0.959
Congestive heart failure	43	9	17.64	34	15.24	0.671
Peripheral vascular disease	7	0	0.0	7	3.13	0.200
Cerebrovascular accident	37	11	21.56	26	11.65	0.062
Dementia	84	17	33.33	67	30.04	0.646
Chronic obstructive pulmonary disease	46	14	27.45	32	14.34	0.024*
Connective tissue disease	1	0	0.0	1	0.44	0.632
Peptic ulcer	8	0	0.0	8	3.58	0.170
Liver disease	6	2	3.92	4	1.79	0.349
Diabetes	52	12	23.52	40	17.93	0.358
Hemiplegia	4	0	0.0	4	1.79	0.335
Chronic kidney disease	14	3	27,45	11	4.93	0.781
Tumor	36	5	9.80	31	13.90	0.435
AMTS (mean ± SD)	7.1 ± 3.6	7.3 ± 3		6.9 ± 3.8		0.522
National Early Warning Score (NEWS) on admission						
0-2	232	44	86.27	188	84.30	0.836
3-6	33	6	11.76	27	12.10	
>6	9	1	1.96	8	3.58	
Time between admission and surgery						
≤36 hours	245	43	84.3	202	90.6	0.021*
>36 hours	31	8	15.7	21	9.4	
Place of residence						
Own home	207	37	17.9	170	82.1	0.551
Care home/intermediate care/nursing home	67	14	20.9	53	79.1	
30-day mortality	33	18	35.29	24	10.72	<0.001*
90-day mortality	61	25	49.01	44	21.97	<0.001*

The mean CCI was 5.2 ± 2.1 in the “positive group” and 4.7 ± 2.2 in the “negative group”; this difference was not statistically significant. A detailed analysis of the CCI components revealed that 27.5% (14/51) of patients in the “positive group” had chronic obstructive pulmonary disease (COPD), whereas only 14.3% (32/223) of patients in the “negative group” had COPD (p = 0.024). The difference was not significant for other comorbidities like myocardial infarction, diabetes, and dementia. Moreover, there were no differences in the mean NHFS and ASA for the positive and negative groups (NHFS, 5.1 ± 1.7 vs. 4.7 ± 1.7; ASA, 3 ± 0.6 vs. 3 ± 0.7; p > 0.05). Most of the patients were hemodynamically stable on admission, with 86.3% and 84.3% of patients in the positive and negative groups, respectively, having National Early Warning Score (NEWS) of 0-2 on admission (Table 1).

Both the groups had a similar proportion of fracture types, with 66.7% of the COVID-19-positive and 70.9% of the COVID-19-negative group having intracapsular fracture in the neck of the femur. Consequently, the proportion of patients undergoing the surgery was similar in both groups; 52.3% and 48.9% of the positive and negative groups undergoing hemiarthroplasty (p > 0.05). Three patients in the positive group and eight in the negative group were managed conservatively as they were unfit for surgery or were bedridden before the injury due to various causes. Further, eight out of the 51 “positive group” patients (15.7%) and 21 of the 223 “negative group” patients (9.4%) experienced a delay in surgery of >36 hours. Interestingly, six of the eight positive patients with delayed surgery died within 90 days postoperatively (Table 1).

The “positive group” group had a prolonged mean hospital stay of 15.7 ± 12.0 days, whereas the “negative group” had a mean hospital stay of 10.1 ± 6.1 days. Moreover, 33 of the 51 positive patients acquired COVID-19 after admission, whereas the remaining 18 were diagnosed as positive on admission. The COVID-19-positive group had increased incidences of respiratory complications and electrolyte imbalance (52.9% and 35.2%, respectively) than the “negative group” (14.8% and 16.2%, respectively) (p < 0.001). There was no difference in the incidence of other complications like myocardial infarction, acute kidney injury (AKI), and neurologic complications (Table 2).

**Table 2:** Comparison of complications between COVID-19-positive and COVID-19-negative groups. Note: * p-value significant at 5% level of significance (p < 0.05).

Complication	Total no. of cases	COVID-19 positive (n = 51)		COVID-19 negative (n = 223)		P-value
		N	%	N	%	
Deep vein thrombosis/embolism	15	5	9.8	10	4.9	0.132
Respiratory complication	49	27	52.9	22	9.8	<0.001*
Cardiac complication	34	10	19.6	24	10.8	0.084
Neurologic complication	17	3	5.9	14	6.3	0.916
Electrolyte imbalance	54	18	35.3	36	16.1	0.002*
Endocrine complications	83	1	1.96	8	3.6	0.149
Acute kidney Injury	34	5	9.8	23	10.31	0.843
Other complication	61	13	25.4	48	21.52	0.387

**Figure 1:**
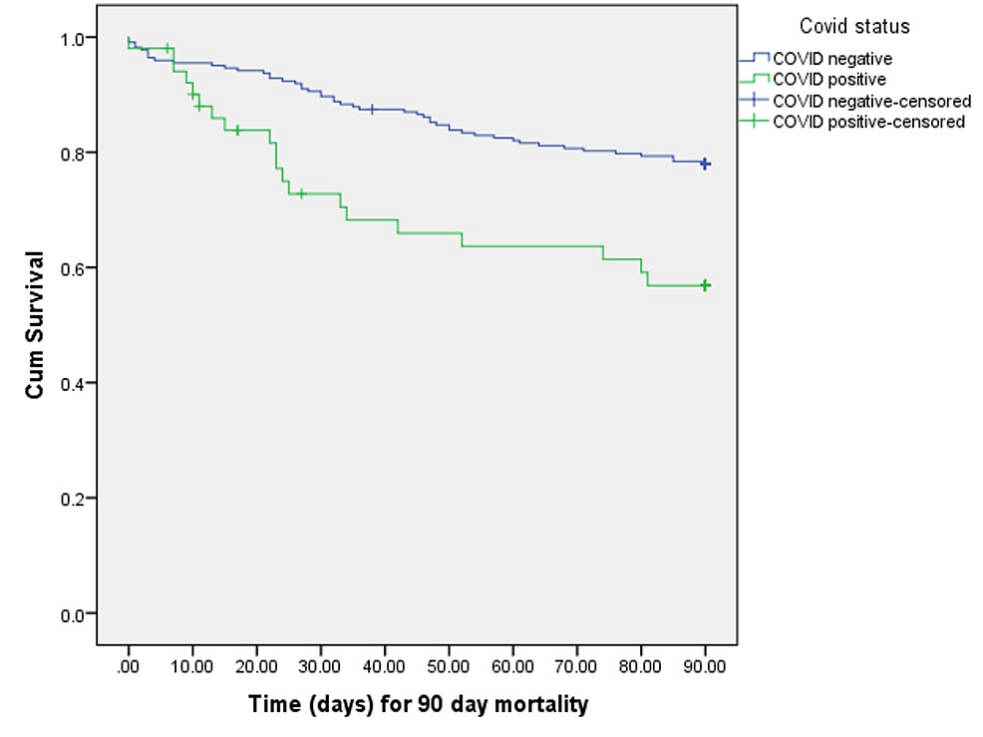
Kaplan-Meier curve showing increased 90-day mortality among COVID-19-positive patients.

The 30- and 90-day mortality rates were significantly higher in the “positive group” (35.2% and 49.01%, respectively) than in the “negative group” (10.72% and 21.97%, respectively) (p < 0.001). Overall, 42 (15.3%) patients died within 30 days and 69 (25.2%) patients died with 90 days of sustaining hip fractures. To eliminate the effect of confounding variables, three separate Cox regression models were used to assess the independent association of COVID-19 and increased mortality risk after adjusting for NHFS, ASA, as well as age and place of residence. COVID-19 was independently associated with an increased risk of 30-day mortality via Cox regression analysis (p < 0.05) (Tables 1, 3) (Figure 1).

**Table 3:** Cox regression models of 30-day survival according to the COVID-19 status. Note: * p-value significant at 5% level of significance (p < 0.05).

Predictors in model	Hazard ratio	95.0% CI		P-value
		Lower	Upper	
Age, sex, place of residence, and COVID-19 status				
No	Reference	-	-	-
Yes	2.056	1.094	3.863	0.025*
Nottingham Hip Fracture Score and COVID-19 status				
No	Reference	-	-	-
Yes	1.981	1.057	3.713	0.033*
American Society of Anesthesiologists (ASA) score and COVID-19 status				
No	Reference	-	-	-
Yes	1.937	1.034	3.631	0.039*

## Discussion

Previous studies have investigated the correlation between COVID-19 in patients with hip fracture and mortality. One such study reported a mortality rate of 40% in a cohort of 10 patients with hip fracture and COVID-19 [13]. Another observational study found high mortality rates among 23 patients with hip fractures and COVID-19 [10]. However, it is difficult to arrive at a conclusion from these studies as the mortality rates were not adjusted to other covariates such as age and pre-existing comorbidities. We aimed to investigate whether COVID-19 alone increases the risk of death after fragility hip fractures. In this study, we determined how COVID-19 affects the mortality and complications in patients with hip fractures. The patients with and without COVID-19 who were admitted to our hospital had similar demographics and NEWS upon admission; thus, only COVID-19 separated the two groups. Later, CCI revealed that the COVID-19-positive group had higher incidences of COPD than the control group; there was no difference in the proportion of other comorbidities between groups.

Early surgery after hip fracture resulted in decreased mortality and complications [12-14]. Catellani et al. showed that early surgery positively impacted the overall outcome of patients with hip fractures and COVID-19 [12]. Best practice guidelines in the UK mandate that surgical intervention for hip fractures should occur within 36 hours of diagnosis. The national mean percentage of patients who underwent surgery within 36 hours in 2018 and 2019 were 69% and 68%, respectively [5,6]. More than 85% of patients in our study underwent surgery within 36 hours; however, delayed surgery was more frequent in the COVID-19-positive group (15.4%) than in the control group (9.4%) (p = 0.021). Unfortunately, six of the eight patients who underwent delayed surgery (>36 hours after diagnosis) died within 90 days of fracture, supporting the fact that early surgery prevents mortality. The reasons for delayed surgery included suboptimal fitness for surgery and staff shortage. Three patients with COVID-19 and eight patients without COVID-19 were treated conservatively; all three patients with COVID-19 died within 90 days.

The IMPACT-Scot 2 report found that more than half of the patients with hip fractures contracted COVID-19 during their hospital stay [15]. In our study, almost two-thirds of patients acquired COVID-19 as an inpatient. Extended hospital stay was a major contributor to increasing the risk of acquiring COVID-19. Kayani et al. observed that the duration of hospital stay for patients with COVID-19 was twice than that of those without COVID-19 [16]. This result is consistent with our study results, wherein the mean duration of hospital stay for patients with COVID-19 was significantly higher (15.7 ± 12.0 days) than that of those without COVID-19 (10.1 ± 6.1 days) (p < 0.05). This was not only due to increased COVID-19-related complications but also due to the constantly changing hospital protocols that delayed patient discharge.

The overall mortality of patients with COVID-19 in literature has varied between 6.9% and 31%; factors such as increased age, comorbidities such as cardiovascular and respiratory diseases, and male sex increased patient mortality [17-22]. According to the National Hip Fracture Database, 30-day mortality for hip fractures was 6.5% in the UK in 2019 [5]. Our study demonstrated that COVID-19 is independently associated with increased 30- and 90-day mortality rates (35.2% and 49.0%) compared with those without COVID-19. Cox regression analyses revealed similar findings, thus eliminating the effects of age, sex, and NHFS. The COVID-19 pandemic has disrupted standard hospital practices. Though the 30- and 90-day mortality rates among patients without COVID-19 (10.72% and 21.97%, respectively) were lower than those of patients with COVID-19, they exceeded the pre-pandemic national mortality rate of 6.5% [5]. In the present study, Kaplan-Meier analysis revealed that the overall 30- and 90-day mortality rates (15.3% and 25.2%, respectively) were higher than the pre-pandemic national average. These differences can be attributed to staff redeployment, paused orthogeriatric services, and reallocation of inpatients to non-orthopedic wards. The results of the present study are consistent with the 30-day mortality identified by other studies in the UK. Hall et al. reported a 30-day mortality of 34.6% for patients with hip fractures and COVID-19 but only 9% for those without COVID-19 [11]. Similarly, Kayani et al. reported 30-day mortality rates of 30.5% and 10.3% for patients with hip fractures and with and without COVID-19 [16], whereas Narang et al. identified 34.9% and 6.1%, respectively [23,24]. To the best of our knowledge, ours is the first study to report 90-day mortality rates in addition to 30-day mortality rates. The 30-day mortality of 35% of patients with COVID-19 increased to 49% at 90 days.

COVID-19 predominantly affects the respiratory system. In this study, patients with COVID-19 had a higher incidence of respiratory complications, such as bacterial pneumonia and acute respiratory distress syndrome, than those without (p < 0.001). The only non-pulmonary complication observed more frequently in COVID-19 positive patients was electrolyte imbalance.

This study has some limitations. Our retrospective study was limited to a single hospital and, therefore, did not account for varied policies and levels of care across facilities. Additionally, we may not have included asymptomatic patients with COVID-19 because the sensitivity of the RT-PCR test is approximately 70% [25,26]. We attempted to minimize this limitation by including those who had clinical or radiological symptoms of COVID-19 as well as by testing patients at regular intervals during their hospital stay.

## Conclusions

COVID-19 is independently associated with increased 30-day and 90-day mortality in hip fracture patients. COVID-19 patients with hip fracture stayed longer in the hospital, had more delayed surgeries, and increased respiratory complications as well as electrolyte imbalance. This study was the first to report the longer-term effects of the COVID-19 pandemic on orthopedic care.
